# Mouse Allergen, Lung Function, and Atopy in Puerto Rican Children

**DOI:** 10.1371/journal.pone.0040383

**Published:** 2012-07-16

**Authors:** Erick Forno, Michelle M. Cloutier, Soma Datta, Kathryn Paul, Jody Sylvia, Deanna Calvert, Sherell Thornton-Thompson, Dorothy B. Wakefield, John Brehm, Robert G. Hamilton, María Alvarez, Angel Colón-Semidey, Edna Acosta-Pérez, Glorisa Canino, Juan C. Celedón

**Affiliations:** 1 Division of Pediatric Pulmonology, Department of Pediatrics, University of Miami, Miami, Florida, United States of America; 2 Department of Pediatrics, University of Connecticut Health Center, Farmington, Connecticut, United States of America; 3 Channing Laboratory, Department of Medicine, Brigham and Women’s Hospital, Boston, Massachusetts, United States of America; 4 Connecticut Children’s Medical Center, Hartford, Connecticut, United States of America; 5 Division of Pediatric Pulmonary Medicine, Allergy and Immunology, Department of Pediatrics, Children’s Hospital of Pittsburgh of UPMC, University of Pittsburgh School of Medicine, Pittsburgh, Pennsylvania, United States of America; 6 Division of Allergy and Immunology, Johns Hopkins University School of Medicine, Baltimore, Maryland, United States of America; 7 Behavioral Sciences Research Institute, University of Puerto Rico, San Juan, Puerto Rico; Ludwig-Maximilians-University Munich, Germany

## Abstract

**Objective:**

To examine the relation between mouse allergen exposure and asthma in Puerto Rican children.

**Methods:**

Mus m 1, Der p 1, Bla g 2, and Fel d 1 allergens were measured in dust samples from homes of Puerto Rican children with (cases) and without (controls) asthma in Hartford, CT (n = 449) and San Juan (SJ), Puerto Rico (n = 678). Linear or logistic regression was used for the multivariate analysis of mouse allergen (Mus m 1) and lung function (FEV_1_ and FEV_1_/FVC) and allergy (total IgE and skin test reactivity (STR) to ≥1 allergen) measures.

**Results:**

Homes in SJ had lower mouse allergen levels than those in Hartford. In multivariate analyses, mouse allergen was associated with higher FEV_1_ in cases in Hartford (+70.6 ml, 95% confidence interval (CI) = 8.6–132.7 ml, P = 0.03) and SJ (+45.1 ml, 95% CI =  −0.5 to 90.6 ml, P = 0.05). In multivariate analyses of controls, mouse allergen was inversely associated with STR to ≥1 allergen in non-sensitized children (odds ratio [OR] for each log-unit increment in Mus m 1 = 0.7, 95% CI = 0.5–0.9, P<0.01). In a multivariate analysis including all children at both study sites, each log-increment in mouse allergen was positively associated with FEV_1_ (+28.3 ml, 95% CI = 1.4–55.2 ml, P = 0.04) and inversely associated with STR to ≥1 allergen (OR for each log-unit increment in Mus m 1 = 0.8, 95% CI = 0.6–0.9, P<0.01).

**Conclusions:**

Mouse allergen is associated with a higher FEV_1_ and lower odds of STR to ≥1 allergen in Puerto Rican children. This may be explained by the allergen itself or correlated microbial exposures.

## Introduction

Mouse allergen (Mus m 1) exposure is common in the mainland United States, where higher allergen levels are found in inner-city or urban areas^[^ [1], [2], [3], [4] ^]^. Published studies of mouse allergen and asthma or asthma morbidity have yielded discrepant results, likely due to differences in study design, analytical approach, allergen levels, and characteristics of study participants.

In a cross-sectional study of 499 inner-city children with asthma in the U.S. mainland, exposure to mouse allergen levels above the median (1.6 µg/g) was associated with mouse allergy but not with asthma morbidity^[^ [5] ^]^. Among 127 preschool children with asthma in Baltimore, those highly exposed to mouse allergen (defined as a level above the lowest quartile or ≥0.5 µg/g) and allergic to mouse had greater asthma morbidity (parental report of symptoms and exacerbations) than those exposed to <0.5 µg/g or those exposed to ≥0.5 µg/g but not allergic to mouse^[^ [6] ^]^. Of note, asthma morbidity in children with high allergen exposure but no mouse allergy was not compared to that of other children.

A cross-sectional study of children (n = 726) and adults (n = 1,643) in the U.S. mainland found that exposure to ≥1.6 µg/g of mouse allergen (a threshold used in a prior study^[^ [5] ^]^) was associated with twofold increased odds of current asthma in subjects with self-reported doctor-diagnosed allergies, even after accounting for exposure to other allergens^[^ [4] ^]^; however, there was no significant association in subjects without self-reported allergies (odds ratio [OR] = 0.7, 95% confidence interval [CI] = 0.3–1.5)^ [^ [4] ^]^. In a birth cohort study of 498 children with parental history of asthma or allergies, detectable mouse allergen at age 2–3 months was not significantly associated with wheeze in the first year of life after adjustment for cockroach allergen^[^ [7] ^]^. In a follow-up study of the same children up to age 7 years, detectable mouse allergen at age 2–3 months was significantly associated with transient wheeze but not with persistent or late-onset wheeze, asthma (OR = 0.6, 95% CI = 0.3–1.3), allergic rhinitis or eczema^[^ [8] ^]^. Although there was a significant association between mouse allergen exposure and skin test reactivity (STR) to ≥1 allergen (OR = 2.0, 95% CI = 1.1–3.7), only 248 (49.8%) of the 498 participants had allergy skin testing at school age. More recently, a birth cohort study of 500 children in the U.S. mainland reported an inverse association between exposure to mouse allergen at age 3 months (analyzed as a continuous log-transformed variable) and single wheeze or allergic sensitization at age 1 year^[^ [9] ^]^.

Puerto Ricans, who are U.S. citizens, bear a disproportionate burden of asthma in this country^[^ [10], [11], [12], [13] ^]^. In spite of high likelihood of mouse allergen exposure among Puerto Ricans in urban areas, there has been no study of mouse allergen and asthma in this ethnic group. In this report, we examine the relation between mouse allergen and asthma, lung function and atopy in a case-control study of Puerto Rican children living in two cities in the U.S. Northeast (Hartford, Connecticut) and the island of Puerto Rico (San Juan).

## Materials and Methods

### Subject Recruitment

A detailed description of the Methods is provided in **Methods S1**.

From September of 2003 to July of 2008, informational flyers were distributed to all parents of children in grades K-8 in 15 public elementary/middle schools in Hartford that enroll a significant proportion (42% to 94%) of Puerto Rican children. Of 640 children whose parents were interested in the study, 585 (91. 4%) were eligible for inclusion after completion of a screening questionnaire; parents of 449 (76.7%) of these 585 children agreed to participate. There were no significant differences in age, gender, or area of residence between eligible children who did (n = 449) and did not (n = 136) agree to participate. Of these 449 children, 427 (95.1%) had data on indoor allergens and were thus included in this analysis.

From March of 2009 to June of 2010, children in San Juan (SJ) were chosen from randomly selected households, using a scheme similar to that of a prior study^[^ [14] ^]^. In brief, households in the Standard Metropolitan Area of SJ were selected by a multistage probability sample design^[^ [14] ^]^. Primary sampling units (PSUs) were randomly selected neighborhood clusters based on the 2000 U.S. census, and secondary sampling units were randomly selected households within each individual PSU. A household was eligible if ≥1 resident was a child 6 to 14 years old. In households with more than one eligible child, a maximum of five children were randomly selected. Within each housing unit selected, children were enumerated and one child per eligible household was selected for screening. In households with multiple eligible children, one child was randomly selected by using Kish tables. On the basis of the sampling design, a total of 7,073 households were selected for inclusion; 6,401 (90.5%) were contacted. Of these 6,401 households, 1,111 had ≥1 child within the age range of the study who met other inclusion criteria (see below). In order to reach our target sample size (∼700 children), we attempted to enroll a random sample (n = 783) of these 1,111 children. Parents of 105 (13.4%) of these 783 eligible households refused to participate or could not be reached. There were no significant differences in age, gender, or area of residence between eligible children who did (n = 678) and did not (n = 105) agree to participate.

In both study sites, the main recruitment tool was a screening questionnaire given to parents of children ages 6 to 14 years to obtain information about the child’s respiratory health and Puerto Rican ancestry. All participants (cases and controls) had to have four Puerto Rican grandparents and be living in the same household for ≥1 year. We selected as cases children with physician-diagnosed asthma and wheeze in the prior year, and as controls children with no physician-diagnosed asthma and no wheeze in the prior year.

### Study Procedures

A description of all study procedures is provided in **Methods S1**. Participants at both sites completed a protocol that included questionnaires, spirometry, allergy skin testing, and collection of blood (for measurement of serum total IgE) and dust samples. Dust samples were obtained from three areas in the home: the one in which the child sleeps (usually his/her bedroom), the living room/television room, and the kitchen. The dust was sifted through a 50-mesh metal sieve, and the fine dust was reweighed, extracted, and aliquoted for analysis of allergens from mouse (mouse urinary protein [Mus m 1]), dust mite (Dermatophagoides pteronyssinus [Der p 1]), cockroach (Blatella germanica [Bla g 2]), and cat dander (Fel d 1) using two-site monoclonal antibody ELISA assays.

### Ethics Statement

Written parental consent was obtained for participating children, from whom written assent was also obtained. The study was approved by the Institutional Review Boards (IRBs) of Connecticut Children’s Medical Center (Hartford [Protocol #135503]), the University of Puerto Rico (SJ [Protocol # 0160507]), Brigham and Women’s Hospital (Boston, MA [Protocol # 2007P-001174), and the University of Pittsburgh (Pittsburgh, PA [Protocol # PRO10030498]).

### Statistical Analysis

Non-detectable allergen levels were assigned a constant (half the lowest detectable value). Allergen levels were analyzed as continuous (after log_10_-transformation). Our outcomes of interest were FEV_1_ and FEV_1_/FVC, total serum IgE, and STR to ≥1 allergen. All analyses were first conducted separately in children with (cases) and without (controls) asthma at each study site. Given results of prior studies, we then conducted analyses stratified by STR to mouse in the combined cohort (including children in Hartford and SJ) because of small sample size at either study site. Univariate analyses were conducted using Fisher exact tests for categorical variables and two-tailed *t* tests for categorical and continuous variables. Linear or logistic regression was used to study the relation between mouse allergen and the outcomes of interest while adjusting for potential confounders. All multivariate models included mouse allergen, age, sex, household income (< vs. ≥$15,000/year [the median household income for Puerto Rico in 2008-2009]), parental (maternal or paternal) history of asthma, and levels of other allergens (cockroach, cat and dust mite); all analyses of FEV_1_ were additionally adjusted for height and height squared. Multivariate analyses of the combined cohort were additionally adjusted for study site. Results with P-values <0.05 were considered significant and those with P values ≥0.05 but <0.10 were considered as of borderline statistical significance. All statistical analysis was performed using SAS statistical software, version 9.2 (SAS Institute; Cary, NC).

## Results

**Table 1** summarizes the main characteristics of study participants. Compared to control subjects at each study site, cases were more likely to have a parental history of asthma, a lower FEV_1_/FVC, higher total IgE, and STR to ≥1 allergen. In SJ, cases were more likely to have low FEV1 and to have STR to dust mite or cockroach than controls; in Hartford, cases were more likely to have STR to cat than controls. At each study site, there was no significant difference in exposure to mouse or any of the other allergens between cases and controls.

**Table 1:** Main characteristics of study participants.

	San Juan, PR		Hartford, CT	
	Cases	Controls	Cases	Controls
N	351	327	267	182
Mean age (yrs)	10.0 (2.6)*	10.5 (2.7)	9.9 (2.8)	9.7 (2.8)
Male gender	57.3%*	48.8%	49.8%	47.3%
Parental asthma:				
• Mother	49.1%*	20.9%	47.0%*	28.1%
• Father	34.05%&	15.5%	35.8*	17.7%
• Either parent	68.1%*	33.2%	66.3%*	44.7%
Parental education:				
• High school or less	45.3%	48.2%	28.5%	26.1%
• At least some college	48.1%	46.6%	64.0%	63.0%
• Missing	6.6%	5.2%	7.5%	10.9%
Household income:				
• <$15,000/year	66.1%	63.2%	56.2%	59.8%
• ≥$15,000/year	33.9%	35.3%	33.3%	29.9%
• Missing	0%	1.5%	10.5%	10.3%
Home allergen level^a^				
• Mus m 1 (ng/g)	6.5 (2.0–31.0)	7.0 (2.0–28.0)	80.6 (15.8–231)	76.8 (27.0–303)
• B.germanica (U/g)	1.30 (.73–4.39)	1.20 (.73–2.75)	1.12 (.30–4.04)	1.58 (.30–4.75)
• Fel d 1 (µg/g)	.02 (.006–.06)*^†^*	.02 (.008–.15)	.36 (.13–1.41)	.25 (.12–1.23)
• Der p (µg/g)	4.51 (2.42–9.57)	4.45 (1.94–9.43)	.15 (.15–.50)	.15 (.15–.56)
				
Spirometry (N)	287	273	266	181
FEV_1_ (liters)	1.90 (0.70)*	2.07 (0.77)	1.92 (0.71)	2.00 (0.71)
FEV_1_/FVC	80.8 (9.1)*	83.0 (9.6)	82.1 (8.7)*	84.7 (9.1)
				
Allergy markers (N)	305	285	257	168
Total IgE (IU/mL)^a^	346 (116–881)*	155 (44–586)	118 (40–348)*	67 (25–303)
				
Positive STR (N)	285	260	267	182
• Mouse	26.2%	21.2%	4.7%	1.4%
• Cockroach	40.1%*	27.5%	21.4%*^†^*	14.3%
• Cat	37.0%	34.0%	24.2%*	12.1%
• Dust mite	55.4%*	41.8%	33.9%	27.9%
• Mold	12.2%	14.0%	4.7%	1.4%
• ≥ 1 allergen	84.2%*	75.0%	47.2%*	35.2%

Mean (SD) for continuous variables, except

a presented as median (IQR), analyzed as log10.

* P-value<0.05 and ^†^P<0.10 for cases vs controls within each group.

STR = skin test reactivity.FEV_1_ shown as absolute value because of lack of predicted values in Puerto Ricans.

**Figure 1:**
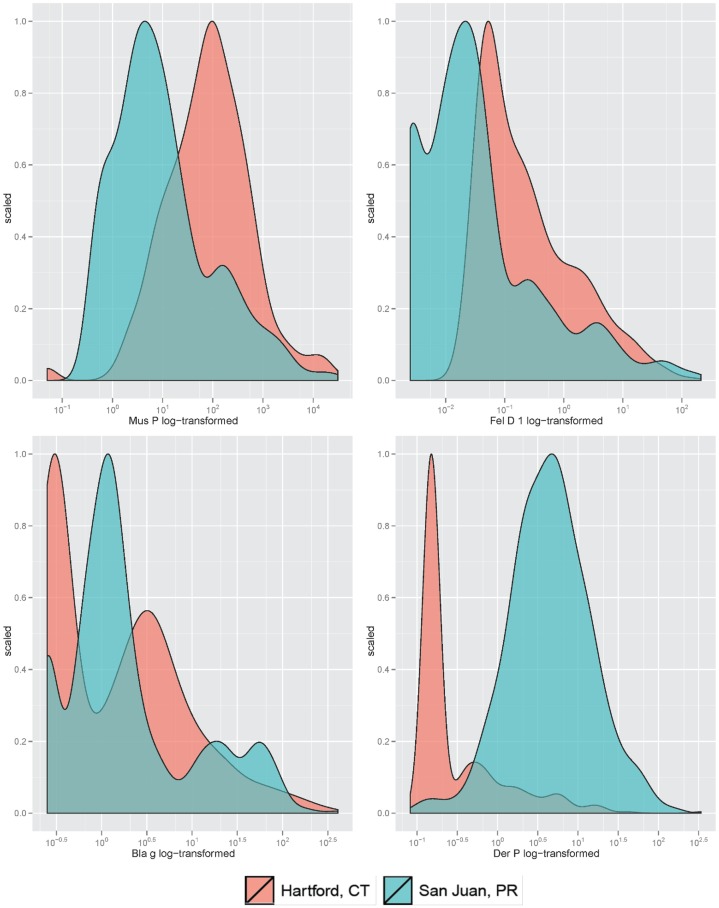
Distribution of allergen levels (log-transformed), by study site.

**Figure 2:**
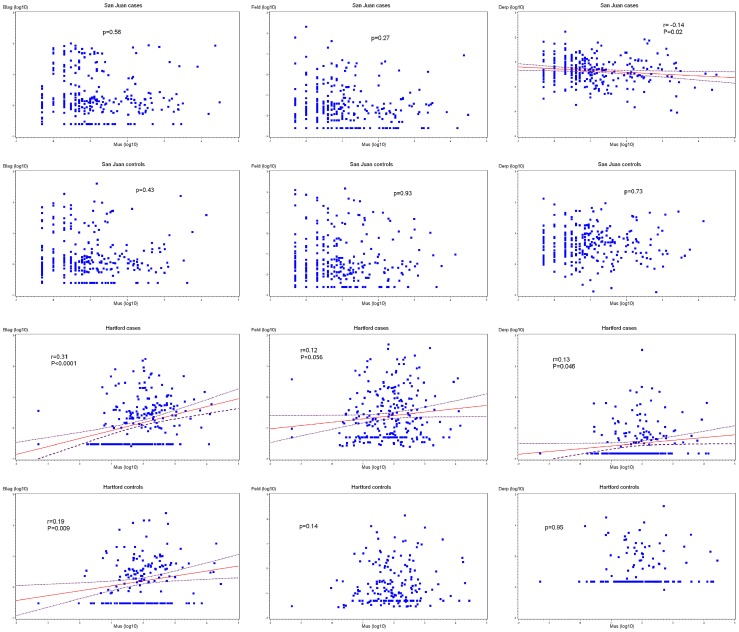
Allergen level correlations in cases and controls, by study site. Correlations with P<0.05 have a regression line and confidence intervals.

Children in SJ (cases or controls) were more likely to have STR to the allergens tested than those in Hartford. Households of children (cases or controls) in SJ had lower levels of mouse and cat allergens but higher levels of dust mite allergen than those of children in Hartford (**Table 1** and **Figure 1**). In Hartford, mouse allergen was weakly but significantly correlated with all other allergens (cockroach, cat, and dust mite) in cases, as well as with cockroach allergen in controls. In SJ, mouse allergen was significantly correlated with dust mite allergen in cases; there was no other significant correlation in cases or controls (**Figure 2**).

Next, we assessed whether allergen exposure influences lung function and atopy in children with asthma (cases). There were slight differences in mouse or cockroach allergen level but otherwise no significant differences between cases with and without spirometry or allergy skin testing (AST) in SJ (**Table S1**). All cases in Hartford had AST, and only one did not undergo spirometry.

**Table 2** shows the results of the unadjusted analysis of quintiles of mouse allergen, FEV_1_ and allergy markers in cases. In SJ, mouse allergen level was significantly and linearly associated with higher FEV_1_ and lower prevalence of STR to cockroach. In Hartford, mouse allergen level was linearly associated with higher FEV_1_ (P = 0.10). There was no significant association between mouse allergen and FEV_1_/FVC at either study site. **Table 3** shows the results of the multivariate analyses of mouse allergen and the outcomes of interest in cases. After adjusting for exposure to other allergens and other covariates, mouse allergen was associated with higher FEV_1_ in SJ and in Hartford, and with lower total IgE in SJ (P≤0.05 in all instances). In this multivariate analysis, dust mite allergen was significantly associated with lower FEV_1_ and STR to ≥1 allergen in Hartford.

**Table 2:** Mouse allergen level, selected covariates, and measures of lung function and atopy in children with asthma.

Quintile of mouse allergen level	Q1	Q2	Q3	Q4	Q5	P for trend
Range (ng/g)	(.05–2.86)	(3.0–10.0)	(10.2–42.0)	(42.02–183.6)	(184–31,227)	
	**SAN JUAN**					
N (%)	83 (28.4)	91 (31.2)	50 (17.1)	27 (9.3)	41 (14.0)	
Mean age (yrs)	10.1 (2.5)	9.7 (2.6)	10.1 (2.7)	9.8 (2.8)	10.8 (2.6)	0.20
Male gender	60%	55%	61%	64%	56%	0.99
Parental asthma	67%	74%	63%	78%	68%	0.90
Parental education beyond high school	52%	48%	43%	61%	59%	0.37
Household income ≥$15,000/year	31%	36%	30%	15%	29%	0.28
FEV_1_ (liters)*	1.86 (0.65)	1.82 (0.69)	1.85 (0.69)	1.92 (0.79)	2.18 (0.72)	**0.02**
FEV_1_/FVC (%)	81.8 (7.5)	80.5 (8.2)	79.2 (0.12)	80.1 (11.9)	81.9 (6.8)	0.80
Total IgE (IU/ml)^a^	418 (138–778)	360 (130–890)	195 (59–668)	397 (86–830)	218 (48–867)	**0.06**
STR(+) to:						
• Mouse	31%	28%	23%	21%	21%	0.18
• Cockroach	51%	40%	34%	22%	29%	**0.007**
• Cat	42%	40%	29%	28%	32%	0.14
• Dust mite	61%	56%	47%	48%	47%	**0.09**
• Mold	12%	15%	17%	9%	9%	0.70
• ≥ 1 allergen	87%	84%	83%	72%	82%	0.23
	**HARTFORD**					
N (%)	13 (5.0)	29 (11.1)	56 (21.4)	91 (34.9)	72 (27.6)	
Mean age (yrs)	9.5 (2.7)	8.8 (2.3)	10.2 (2.8)	10.0 (2.7)	10.0 (3.1)	0.19
Male gender	62%	38%	61%	46%	47%	0.52
Parental asthma	62%	71%	57%	68%	72%	0.31
Parental education beyond high school	62%	59%	61%	70%	61%	**0.09**
Household income ≥$15,000/year	46%	52%	45%	29%	24%	**0.0003**
FEV_1_ (liters)*	1.76 (0.50)	1.69 (0.57)	1.94 (0.66)	1.96 (0.73)	1.99 (0.78)	0.10
FEV_1_/FVC (%)	82.4 (10.6)	80.2 (7.6)	83.2 (7.0)	82.0 (9.4)	82.4 (9.0)	0.99
Total IgE (IU/ml)^a^	387 (55–345)	109 (24–239)	92 (31–220)	115 (56–345)	146 (51–411)	0.43
STR(+) to:						
• Mouse	0%	8%	2%	8%	2%	0.79
• Cockroach	20%	12%	19%	23%	24%	0.26
• Cat	20%	24%	19%	28%	24%	0.61
• Dust mite	40%	28%	29%	35%	36%	0.57
• Mold	10%	4%	2%	5%	5%	0.98
• ≥ 1 allergen	62%	52%	45%	45%	47%	0.46

Quintiles (and ranges) calculated using the combined cohort (San Juan and Hartford). Values shown are mean (SD) for continuous variables, except

a presented as median (IQR), analyzed as log10.

STR = skin test reactivity.

* FEV1 presented as absolute value due to lack of predicted values for Puerto Rican children.

**Table 3:** Multivariate analysis of mouse allergen, FEV_1_, and allergy markers in children with asthma.

Predictors included in the model	Pre-bronchodilator FEV1 (mL)^1^	Total serum IgE^1^	STR to at least one allergen^2^
	**SAN JUAN**		
Unadjusted (N)	266	283	264
Mus m 1 (ng/g)	**+93.9 [13.1;174.8] (0.02)**	**−17.7% [−31.3;−1.4] (0.03)**	0.84 [0.62;1.13] (0.25)
Multivariate model (N)	266	283	264
Mus m 1 (ng/g)	**+45.1 [−0.5;90.6] (0.05)**	**−17.5% [−31.2;−1.1] (0.04)**	0.82 [0.60;1.12] (0.20)
Fel d 1 (µg/g)	−8.3 [−60;43.] (0.75)	−13.4% [−29;6] (0.16)	0.9 [0.6;1.2] (0.43)
Bla g (U/g)	−13.0 [−81;55] (0.71)	−19.7% [−39;5] (0.11)	0.7 [0.4;1.1] (0.12)
Der p (µg/g)	+11.7 [−80;104] (0.80)	+37.8% [−4;99] (0.09)	1.03 [0.5;2.0] (0.92)
	**HARTFORD**		
Unadjusted (N)	260	252	261
Mus m 1 (ng/g)	**+107.8 [5.7;210.0] (0.04)**	+20.8% [−3.7;51.5] (0.10)	0.95 [0.71;1.26] (0.71)
Multivariate model (N)	203	195	204
Mus m 1 (ng/g)	**+70.6 [8.6;132.7] (0.03)**	+10.9 [−17.8;49.6] (0.50)	0.82 [0.55;1.22] (0.32)
Fel d 1 (µg/g)	+54.3 [−7;116] (0.08)	−15.7% [−38;14] (0.26)	0.9 [0.6;1.3] (0.57)
Bla g (U/g)	−20.1 [−86;45] (0.55)	+9.8% [−21;52] (0.57)	1.0 [0.6;1.5] (0.85)
Der p (µg/g)	−128.9 [−212; −46] (0.002)	+45.3% [−2;115] (0.06)	1.9 [1.1;3.2] (0.03)

Values shown are ^1^means or ^2^odds ratios and 95% confidence intervals, with P-values in parentheses. All allergens analyzed as log10. IgE analyzed as log10 and presented as percent increase/decrease. All models adjusted for age, sex, household income, and dust house levels of allergens. FEV1 adjusted additionally for height and height squared.

Given results from previous studies and consistent results across sites in the current study, we repeated the analysis after stratification by STR to mouse in the combined cohort to maximize our statistical power (**Table 4** **).** Among cases sensitized to mouse, there was no significant association between mouse allergen and any outcome (P≥0.75 in all instances). Among cases not allergic to mouse, mouse allergen was associated with higher FEV_1_ (P = 0.07).

**Table 4:** Multivariate analysis of mouse allergen level and selected outcomes in cases, by skin test reactivity to mouse.

Predictors included in the model	Pre-bronchodilator FEV1 (mL)^1^	Total serum IgE^1^	STR to at least one allergen^2,3^
	**STR(+) to mouse**		
Unadjusted (N)	69	70	n/a
Mus m 1 (ng/g)	−41.3 [−197.8;115.3] (0.61)	+1.6% [−27.4;42.3] (0.92)	
Multivariate model (N)	66	67	n/a
Mus m 1 (ng/g)	−13.7 [−99.9;72.5] (0.76)	−5.8% [−35.0;36.6] (0.75)	
Fel d 1 (µg/g)	+4.2 [−88;97] (0.93)	−10.4% [−39;31] (0.57)	
Bla g (U/g)	+3.2 [−94;158] (0.62)	−31.4% [−60;18] (0.17)	
Der p (µg/g)	−10.2 [−181;161] (0.91)	+22.1% [−39;146] (0.58)	
	**STR(–) to mouse**		
Unadjusted (N)	358	369	372
Mus m 1 (ng/g)	**+94.8 [27.1;162.5] (0.006)**	−**14.5% [**−**27.5;1.0] (0.06)**	**0.77 [0.62;0.95] (0.017)**
Multivariate model (N)	317	328	331
Mus m 1 (ng/g)	**+34.4 [**−**2.5;71.3] (0.07)**	−6.6% [−22.5;12.7] (0.48)	0.85 [0.65;1.11] (0.22)
Fel d 1 (µg/g)	+26.0 [−16;68] (0.22)	−14.1% [−31;6] (0.16)	0.8 [0.6;1.1] (0.11)
Bla g (U/g)	+12.8 [−36;62] (0.61)	−13.8% [−33;11] (0.25)	0.8 [0.6;1.1] (0.21)
Der p (µg/g)	−57.5 [−120;5] (0.07)	+41.1% [2;95] (0.04)	1.4 [0.9;2.2] (0.17)

Values shown are ^1^means or ^2^odds ratios and 95% confidence intervals, with P-values in parentheses. All allergens analyzed as log10. IgE analyzed as log10 and presented as percent increase/decrease. ^3^All children allergic to mouse were also sensitized to ≥1 additional allergen. All models adjusted for age, sex, household income, other allergens, and study site. FEV1 adjusted additionally for height and height squared.

Use of inhaled steroids and use of leukotriene receptor antagonists were reported by parents of 34.5% (31.9% in San Juan, 37.5% in Hartford, P = 0.17) and 21.2% (23.9% in San Juan, 18.0% in Hartford, P = 0.09) of cases. In the combined cohort, use of inhaled steroids was associated with lower FEV_1_ (P = 0.09) and use of leukotriene receptor antagonists (LTRA) was associated with higher total IgE (P = 0.01). We obtained similar results after additional adjustment of our multivariate analysis for use of controller medications (data not shown).

We then examined the relation between mouse allergen and the outcomes of interest in children without asthma (controls). In SJ, controls without spirometry had lower total IgE than those with spirometry **(Table S1)**. All controls in Hartford had AST, and only one did not have spirometry. **Table S2 (Online Supplement)** shows the results of the analysis of quintiles of mouse allergen and the outcomes of interest in controls. In SJ, there was no significant association between mouse allergen and any of the outcomes. In Hartford, mouse allergen was significantly associated with lower total IgE and lower odds of STR to dust mite or to ≥1 allergen. **Table S3** shows the results of the multivariate analyses of mouse allergen and the outcomes of interest in controls. In this analysis, mouse allergen was associated with higher FEV_1_ in SJ, and with lower total IgE in Hartford (P<0.10 in both instances). In an analysis stratified by STR to mouse in all control subjects, mouse allergen was associated with lower total IgE (P = 0.10) and lower odds of STR to ≥1 allergen (P<0.01) only among children not allergic to mouse (**Table S4a, Online Supplement**).

We then conducted an analysis stratified by STR to mouse in all children (cases and controls) at each study site and for the combined cohort (**Table S4b, Online Supplement)**. In the multivariate analysis of the combined cohort (adjusting for case-control status, study site, allergen levels and other covariates) mouse allergen was significantly associated with higher FEV_1_ and lower odds of STR to ≥1 allergen (P<0.01 in both instances) in children not sensitized to mouse.

## Discussion

To our knowledge, this is the first study of mouse allergen exposure and lung function. This is also the first study of mouse allergen exposure and asthma or asthma morbidity in Puerto Ricans.

Among cases, we found that mouse allergen was associated with higher FEV_1_ in Hartford and SJ, and that this association remained significant only in children not sensitized to mouse. Among controls, mouse allergen was associated with lower odds of STR to ≥1 allergen in children not sensitized to mouse. In an analysis including all participants (Table S4b), mouse allergen was significantly associated with higher FEV_1_ and lower odds of STR to ≥1 allergen in children not sensitized to mouse.

Our overall findings are interesting and partly consistent with a recent report of an inverse association between exposure to mouse allergen at age 3 months and single wheeze or allergic sensitization at age 1 year^[^ [9] ^]^. Although additional replication is needed, our results suggest that mouse allergen or a factor correlated with this exposure is associated with a higher FEV_1_ and reduced intensity of allergic responses in Puerto Rican children. Previous studies have shown that Hispanic (including Puerto Rican) children with/at risk for asthma are less likely to have cats or dogs^[^ [15], [16] [17] ^]^, and thus mouse allergen may be a marker of increased exposure to microbial components in mammalian feces (endotoxin or muramic acid^[^ [18], [19] ^]^) in Puerto Rican children.

In our study, children living in Hartford were exposed to higher levels of mouse allergen but lower levels of dust mite allergen than those living in SJ. Consistent with prior findings in inner-city children in the U.S. Northeast^[^ [5], [6] ^]^, mouse allergen levels in Hartford were significantly correlated with cockroach allergen in cases and controls (r = 0.20–0.30, P<0.05). In contrast, there was no significant correlation between mouse allergen and cockroach allergen in cases or controls living in a tropical environment (SJ). Unlike participants in prior studies of inner-city children in the U.S. Northeast, most participants in Hartford were not allergic to mouse, which may be explained by attenuated humoral (IgE) responses in children exposed to high levels of mouse allergen^[^ [20] ^]^. Additional differences between our study and previous reports include sample size^[^ [1], [2], [3], [5], [6], [7], [8] ^]^, ethnicity of the participants^[^ [1], [2], [3], [4], [5], [6], [7], [8], [9] ^]^, level of exposure to mouse allergen^[^ [1], [2], [3], [4], [5], [6], [7], [8], [9] ^]^, and analytical approach^[^ [1], [2], [3], [4], [5], [6], [7], [8] ^]^ (all studies showing detrimental or no effects of mouse allergen on asthma morbidity mainly assessed mouse allergen as a categorical variable).

The only previous study of allergen exposure and asthma in Puerto Rico included ∼82–89 children with asthma (ages 1 to 17 years) living in the city of Bayamón^[^ [17] ^]^. Compared to our findings for cases in SJ (**Table 1**), that study reported similar average levels of Der p 1 or Der f 1 (4.3 µg/g) but lower average levels of Bla g 2 (0.75 U/g) in house dust (only collected from the child’s mattress and bedside floor); there was no assessment of Mus m 1. In bivariate analyses, none of the allergens included in the current study was significantly associated with asthma symptoms. Although those results could be explained by low statistical power, they are consistent with our negative findings for allergens other than mouse in SJ. As speculated in that prior study, dust mite or cockroach allergen level in house dust may not adequately reflect airborne allergen exposure in Puerto Rico (where homes may be more ventilated homes than in the U.S. Northeast). Alternatively, there may be differences in other (unmeasured) environmental exposures across study sites such as indoor endotoxin or fungal allergens, or outdoor air pollution.

Our findings for dust mite allergen and FEV_1_ in Hartford are consistent with those of Gent *et al*., who found a significant association between Der p 1 levels ≥2 µg/g and increased asthma severity (assessed by questionnaire) over 1 year of follow-up in 300 children (ages 4 to 12 years) with asthma (18.3% of whom were Hispanic) in the U.S. Northeast (including Connecticut)^ [^ [21] ^]^. Consistent with our results in Hartford but in contrast to a prior report in inner-city U.S. children^[^ [22] ^]^, Gent et al. found no significant association between cockroach allergen and asthma severity^[^ [21] ^]^.

Adjustment of the multivariate analysis of Mus m 1 and the outcomes of interest for Der p 1 and other allergens did not change the direction of the observed associations but sometimes led to marked changes in the magnitude of such associations (e.g., see the unadjusted and adjusted results for Mus m 1 and FEV_1_ in Table 3). This stresses the importance of accounting for other allergens in future studies of Mus m 1.

We recognize several limitations to our findings. Firstly, selection bias is possible in any observational study, particularly one of ethnic minorities. However, selection bias is an unlikely explanation for our results in general and in SJ in particular. Children in SJ were randomly sampled, and most (∼87%) eligible children participated in the study. Among participating children in Hartford or SJ, there were no marked differences in allergen levels or other covariates between children who did and did not complete key study procedures (spirometry or AST). Secondly, we cannot assess the effects of allergen exposure in early life on asthma or allergy in a cross-sectional study of children of school age. However, a cross-sectional study of allergen exposure and lung function is valid and can yield useful information. Thirdly, we are not able to adequately examine mouse allergen and lung function or atopy in children sensitized to mouse because of small sample size for that subgroup. Lastly, conducting subgroup analyses increases the risk of false positive findings. However, the observed associations forFEV_1_ and STR to ≥1 allergen were consistent in direction (positive for FEV_1_ and negative for STR to ≥1 allergen) across study sites and asthma status, and they were significant among all children not sensitized to mouse after adjustment for potential confounders. Differences in the magnitude or degree of significance across study sites or among subgroups may at least partly be due to variations in the distribution of mouse allergen and/or sample size.

In summary, mouse allergen exposure was associated with higher FEV_1_ and lower odds of STR to ≥1 allergen in Puerto Rican children. Our findings merit further assessment of factors that could mediate this association (e.g., microbial exposures associated with the presence of mice).

## Supporting Information

Methods S1 S1. Subject recruitment. S2. Study Procedures.
(DOC)

Table S1 Mean (SD) for continuous variables, except ^a^presented as median (IQR), analyzed as log10. *P<0.05 for comparison within each outcome. STR = skin test reactivity.
(DOC)

Table S2 Quintiles (and ranges) calculated using the combined cohort (San Juan and Hartford). Values shown are mean (SD) for continuous variables, except ^a^presented as median (IQR), analyzed as log10. STR = skin test reactivity. *FEV1 presented as absolute value due to lack of predicted values for Puerto Rican children.
(DOC)

Table S3 Values shown are ^1^means or ^2^odds ratios and 95% confidence intervals, with P-values in parentheses. All allergens analyzed as log10. IgE analyzed as log10 and presented as percent increase/decrease. All models adjusted for age, sex, household income, and dust house levels of allergens. FEV1 adjusted additionally for height and height squared.
(DOC)

Table S4 Table S4a: Values shown are ^1^means or ^2^odds ratios and 95% confidence intervals, with P-values in parentheses. ^3^All children with STR to mouse also had STR to ≥ additional allergen. All allergens analyzed as log10. IgE analyzed as log10 and presented as percent increase/decrease. All models adjusted for age, sex, household income, dust house levels of allergens, and study site. FEV1 adjusted additionally for height and height squared. Table S4b, part A: Values shown are ^1^means or ^2^odds ratios and 95% confidence intervals, with P-values in parentheses. ^3^All children with STR to mouse also had STR to ≥1 additional allergen. All allergens analyzed as log10. IgE analyzed as log10 and presented as percent increase/decrease. All models adjusted for age, sex, household income, dust house levels of allergens, and disease status (case or control). FEV1 adjusted additionally for height and height squared. Unable to conduct multivariate analyses in Hartford. Table S4b, part B: Values shown are ^1^means or ^2^odds ratios and 95% confidence intervals, with P-values in parentheses. All allergens analyzed as log10. IgE analyzed as log10 and presented as percent increase/decrease. All models adjusted for age, sex, household income, dust house levels of allergens, and disease status (case or control). FEV1 adjusted additionally for height and height squared. Table S4b, part C: Values shown are ^1^means or ^2^odds ratios and 95% confidence intervals, with P-values in parentheses. ^3^All children with STR to mouse also had STR to ≥1 additional allergen. All allergens analyzed as log10. IgE analyzed as log10 and presented as percent increase/decrease. All models adjusted for age, sex, household income, dust house levels of allergens, study site, and disease status (case or control). FEV1 adjusted additionally for height and height squared.
(DOC)
