# Supplementary material for: Mouse Allergen, Lung Function, and Atopy in Puerto Rican Children
Source: PLoS One. 2012 Jul 16;7(7):e40383. doi: 10.1371/journal.pone.0040383 (PMC3398035; doi:10.1371/journal.pone.0040383)
Supplement: Methods S1 — S1. Subject recruitment. S2. Study Procedures. (DOC) [file pone.0040383.s001.doc]

**ONLINE SUPPLEMENT**

**Mouse Allergen, Lung Function, and Atopy in Puerto Rican Children**

Erick Forno, M.D., M.P.H.^1^, Michelle M. Cloutier, M.D.^2^, Soma Datta^3^, Kathryn Paul^3^, Jody Sylvia, M.S.^3^, Deanna Calvert^4^; Sherell Thornton-Thompson^4^; Dorothy B. Wakefield, M.A.^2^, John Brehm, M.D., M.P.H.^5^, Robert G. Hamilton^6^, María Alvarez, M.D.^7^, Angel Colón-Semidey, M.D.^7^, Edna Acosta-Pérez, Ph.D.^7^, Glorisa Canino, Ph.D.^7^, Juan C. Celedón, M.D., Dr.P.H., F.C.C.P.^5*^

^1^Division of Pediatric Pulmonology, Dept. of Pediatrics, University of Miami, Miami, FL;^2^Department of Pediatrics, University of Connecticut Health Center, Farmington, CT;

^3^Channing Laboratory, Dept. of Medicine, Brigham and Women’s Hospital, Boston, MA; ^4^Connecticut Children’s Medical Center, Hartford, CT; ^5^Division of Pediatric Pulmonary Medicine, Allergy and Immunology, Dept. of Pediatrics, Children’s Hospital of Pittsburgh of UPMC, University of Pittsburgh School of Medicine, Pittsburgh, PA; ^6^Division of Allergy and Immunology, Johns Hopkins University School of Medicine, and ^7^Behavioral Sciences Research Institute, University of Puerto Rico, San Juan, Puerto Rico.

*Corresponding author: Juan C. Celedón, M.D., Dr.P.H.

Division of Pediatric Pulmonary Medicine,

Allergy and Immunology, Dept. of Pediatrics

Children’s Hospital of Pittsburgh of UPMC

4401 Penn Avenue, Pittsburgh, PA 15224

Phone: 412.692.8429; Fax: 412.692.7636

Email: juan.celedon@chp.edu

**Sources of support:** Grant HL079966 from the U.S. National Institutes of Health and General Clinical Research Center at the University of Connecticut Health Center

**METHODS**

**S1. Subject recruitment**

From September of 2003 to July of 2008, informational flyers were distributed to all parents of children in grades K-8 in 15 public elementary/middle schools in Hartford that enroll a significant proportion (42% to 94%) of Puerto Rican children. Of 640 children whose parents were interested in the study, 585 (91.4%) were eligible for inclusion after completion of a screening questionnaire; parents of 449 (76.7%) of these 585 children agreed to participate. Of these 449 children, 95 (21.2%) were recruited between September of 2003 and October of 2005, and 354 (78.8%) were recruited between September of 2006 (following funding from the National Institutes of Health) and July of 2008. There were no significant differences in age, gender, or area of residence between eligible children who did (n=449) and did not (n=136) agree to participate.

From March of 2009 to June of 2010, children in San Juan (SJ) were chosen from randomly selected households, using a scheme similar to that of a prior study [1]. In brief, households in the Standard Metropolitan Area of SJ were selected by a multistage probability sample design [1]. Primary sampling units (PSUs) were randomly selected neighborhood clusters based on the 2000 U.S. census, and secondary sampling units were randomly selected households within each individual PSU. A household was eligible if ≥1 resident was a child 6 to 14 years old. In households with more than one eligible child, a maximum of five children were randomly selected. Within each housing unit selected, children were enumerated and one child per eligible household was selected for screening. In households with multiple eligible children, one child was randomly selected by using Kish tables. On the basis of the sampling design, a total of 7,073 households were selected for inclusion; 6,401 (90.5%) were contacted. Of these 6,401 households, 1,111 had ≥1 child within the age range of the study who met other inclusion criteria (see below). In order to reach our target sample size (~700 children), we attempted to enroll a random sample (n=783) of these 1,111 children. Parents of 105 (13.4%) of these 783 eligible households refused to participate or could not be reached. There were no significant differences in age, gender, or area of residence between eligible children who did (n=678) and did not (n=105) agree to participate.

In both study sites, the main recruitment tool was a screening questionnaire given to parents of children ages 6 to 14 years to obtain information about the child’s respiratory health and PR ancestry. All participants (cases and controls) had to have four PR grandparents and be living in the same household for ≥1 year. We selected as cases children with physician-diagnosed asthma and wheeze in the prior year, and as controls children with no physician-diagnosed asthma and no wheeze in the prior year.

**S2. Study Procedures**

The parents of each participant completed a questionnaire used in the Genetics of Asthma in Costa Rica Study [2]. Spirometry was conducted with an EasyOne (ndd Medical Technologies, Andover, MA) spirometer following American Thoracic Society recommendations [3]. All subjects had to be free of respiratory illnesses for at least 4 weeks before spirometry, and they were also instructed to avoid use of inhaled short- and long-acting bronchodilators for at least 4 and 12 hours before testing, respectively. The best FEV_1_ and FVC were selected for data analysis of FEV_1_ and FEV_1_/FVC.

Dust samples were obtained from three areas in the home: the one in which the child sleeps (usually a bedroom), living room/television room, and kitchen. The dust was sifted through a 50-mesh metal sieve, and the fine dust was reweighed, extracted, and aliquoted for analysis of allergens from mouse urinary protein (Mus m 1), Dermatophagoides pteronyssinus (Der p 1), Blatella germanica (Bla g 2), and cat dander (Fel d 1) using monoclonal antibody Multiplex array assays using the same reagents employed in the established ELISA[4]. Internal controls were run in each assay to insure inter-assay reproducibility.

Serum total IgE was measured using the UniCAP 100 system (Pharmacia & Upjohn, Kalamazoo, MI). Skin test reactivity (STR) to aeroallergens was assessed using a Multi Test device (Lincoln Diagnostics, Decatur, IL) in a site free of eczema: in addition to histamine (positive control) and saline solution (negative control), allergen extracts from house dust mite mix (Dermatophagoides pteronyssinus and Dermatophagoides farinae), German cockroach (Blatella germanica), cat dander, dog dander, mixed grass pollen, mugwort sage, ragweed, mixed tree pollen, mold mix, Alternaria tenuis and mouse pelt were applied to the skin of the forearm (Alk-Abello, Round Rock, TX). A test was considered positive if the maximum diameter of the wheal was > 3 mm after subtraction of the maximum diameter of the negative control.

***REFERENCES***

1. Bird HR, Canino GJ, Davies M, Duarte CS, Febo V, et al. (2006) A study of disruptive behavior disorders in Puerto Rican youth: I. Background, design, and survey methods. J Am Acad Child Adolesc Psychiatry 45: 1032-1041.

2. Hunninghake GM, Soto-Quiros ME, Avila L, Ly NP, Liang C, et al. (2007) Sensitization to Ascaris lumbricoides and severity of childhood asthma in Costa Rica. J Allergy Clin Immunol 119: 654-661.

3. (1995) Standardization of Spirometry: 1994 Update. Am J Respir Crit Care Med 152: 1107-1136.

4. Luczynska CM, Arruda LK, Platts-Mills TA, Miller JD, Lopez M, et al. (1989) A two-site monoclonal antibody ELISA for the quantification of the major Dermatophagoides spp. allergens, Der p I and Der f I. J Immun Methods 118: 227-235.
