# Supplementary material for: Mouse Allergen, Lung Function, and Atopy in Puerto Rican Children
Source: PLoS One. 2012 Jul 16;7(7):e40383. doi: 10.1371/journal.pone.0040383 (PMC3398035; doi:10.1371/journal.pone.0040383)
Supplement: Table S1 — Mean (SD) for continuous variables, except apresented as median (IQR), analyzed as log10. *P<0.05 for comparison within each outcome. STR = skin test reactivity. (DOC) [file pone.0040383.s002.doc]

***Table S1:*** ***Comparison of Cases with and without Spirometry or Allergy Skin Testing***

**A1) San Juan – Cases**

|  | **Spirometry** | | **Allergy skin testing** | |
| --- | --- | --- | --- | --- |
|  | **With** | Without | **With** | Without |
| N | 287 | 64 | 285 | 66 |
| Mean age (yrs) | 10.0 (2.6) | 10.0 (2.7) | 10.0 (2.6) | 10.0 (2.6) |
| Male gender | 57.4% | 56.3% | 58.3% | 53.0% |
| Parental asthma:  Mother  Father  Either parent | 49.5%  33.5%  69.1% | 47.6%  36.7%  63.9% | 48.1%  35.6%  69.2% | 51.9%  27.4%  63.9% |
| Parental education:  High school or less  More than high school  N/A | 42.2%  49.8%  8.0% | 56.3%  40.6%  3.1% | 43.5%  49.1%  7.4% | 43.9%  53.0%  3.1% |
| Household income:  <$15,000/year  ≥$15,000/year  N/A | 66.6%  33.4%  0% | 64.1%  33.9%  0% | 68.4%  31.5%*  0% | 56.1%  43.9%*  0% |
| Home allergen level^a^  Mus m 1 (ng/g)  B.germanica (U/g)  Fel d 1 (μg/g)  Der p (μg/g) | 7.0 (2.0-42.0)*  1.38 (.73-4.53)  .02 (.006-.06)  4.5 (2.4-9.6) | 3.5 (1.0-7.0)  1.22 (.65-1.88)  .02 (.007-.05)  4.9 (2.6-8.4) | 6.5 (2.0-32.0)  1.28 (.69-4.01)*  .02 (.007-.06)  4.5 (2.3-9.6) | 6.5 (2.0-17.5)  1.96 (1.0-41.4)  .02 (.004-.03)  5.8 (3.3-11.3) |
| Spirometry |  |  |  |  |
| Baseline FEV_1_ (liters) |  |  | 1.90 (0.69) | 1.97 (0.77) |
| Baseline FEV_1_/FVC |  |  | 80.6 (9.1) | 83.8 (9.3) |
| Atopy markers |  |  |  |  |
| Eosinophils (cells/μL)^a^ | 397 (227-608) | 314 (209-555) | 395 (237-611) | 286 (113-522) |
| Total IgE (IU/mL)^a^ | 330 (115-815) | 598 (140-1133) | 361 (117-881) | 264 (62-887) |
| Allergy skin testing |  |  |  |  |
| STR to: Mouse  Cockroach  Cat  Dust mite  Mold  ≥1 allergen | 26%  38%*  37%  55%  12%  84% | 30%  60%  40%  55%  10%  90% |  |  |

Mean (SD) for continuous variables, except ^a^presented as median (IQR), analyzed as log10.*****P <0.05 for comparison within each outcome. STR=skin test reactivity.

**A2) San Juan – Controls**

|  | **Spirometry** | | **Allergy skin testing** | |
| --- | --- | --- | --- | --- |
|  | **With** | Without | **With** | Without |
| N | 270 | 57 | 261 | 66 |
| Mean age (yrs) | 10.5 (2.7) | 10.9 (2.9) | 10.6 (2.7) | 9.9 (2.7) |
| Male gender | 51.9% | 49.1% | 46.7% | 56.1% |
| Parental asthma:  Mother  Father  Either parent | 20.6%  15.2%  32.6% | 21.8%  16.9%  35.9% | 21.6%  14.7%  32.5% | 17.4%  18.7%  35.6% |
| Parental education:  High school or less  More than high school  N/A | 48.9%  45.2%  5.9% | 52.6%  45.6%  1.8% | 51.2%*  43.4%  5.3% | 43.6%  48.7%  7.7% |
| Household income:  <$15,000/year  ≥$15,000/year  N/A | 64.1%  34.4%  1.5% | 57.9%  40.4%  1.8% | 63.9%  35.0%  1.1% | 60.6%  36.4%  3.0% |
| Home allergen level^a^  Mus m 1 (ng/g)  B.germanica (U/g)  Fel d 1 (μg/g)  Der p (μg/g) | 6.0 (2.0-28.0)  1.18 (.73-3.08)  .02 (.008-.11)  4.4 (2.0-9.5) | 7.0 (4.0-24.0)  1.20 (.25-1.73)  .02 (.01-.66)  4.4 (1.9-7.7) | 7.0 (2.0-26.0)  1.15 (.73-2.73)  .02 (.008-.15)  4.4 (1.9-9.5) | 6.0 (2.0-30.0)  1.43 (.68-3.60)  .04 (.008-.13)  5.1 (2.3-7.0) |
| Spirometry |  |  |  |  |
| Baseline FEV_1_ (liters) |  |  | 2.07 (0.75) | 1.84 (0.65) |
| Baseline FEV_1_/FVC |  |  | 83.4 (8.9) | 84.5 (8.8) |
| Atopy markers |  |  |  |  |
| Eosinophils (cells/μL)^a^ | 231 (133-416)* | 138 (83-239) | 228 (129-391) | 195 (129-540) |
| Total IgE (IU/mL)^a^ | 167 (46-620)* | 51 (29-249) | 153 (45-586) | 182 (33-693) |
| Allergy skin testing |  |  |  |  |
| STR to: Mouse  Cockroach  Cat  Dust mite  Mold  Any STR(+) | 22.1%  28.8%  33.0%  41.5%  15.6%  74.2% | 10.5%  15.8%  42.9%  42.9%  0%  85.7% |  |  |

Mean (SD) for continuous variables, except ^a^presented as median (IQR), analyzed as log10.*****P <0.05 for comparison within each outcome. STR=skin test reactivity.
