# Supplementary material for: Mouse Allergen, Lung Function, and Atopy in Puerto Rican Children
Source: PLoS One. 2012 Jul 16;7(7):e40383. doi: 10.1371/journal.pone.0040383 (PMC3398035; doi:10.1371/journal.pone.0040383)
Supplement: Table S2 — Quintiles (and ranges) calculated using the combined cohort (San Juan and Hartford). Values shown are mean (SD) for continuous variables, except apresented as median (IQR), analyzed as log10. STR = skin test reactivity. *FEV1 presented as absolute value due to lack of predicted values for Puerto Rican children. (DOC) [file pone.0040383.s003.doc]

***Table S2. Mouse allergen level, selected covariates, and measures of lung function and atopy in controls***

1. San Juan

| **Quintile of mouse allergen level**  Range (ng/g)  N (%) | **Q1**  (.05-2.86)  85 (30.0) | **Q2**  (3.0-10.0)  86 (30.4) | **Q3**  (10.2-42.0)  56 (19.8) | **Q4**  (42.02-183.6)  26 (9.2) | **Q5**  (184-31,227)  30 (10.6) | **P for trend** |
| --- | --- | --- | --- | --- | --- | --- |
| Mean age (yrs) | 10.8 (2.5) | 10.4 (2.9) | 10.8 (2.8) | 10.5 (2.7) | 10.1 (2.8) | 0.37 |
| Male gender | 49% | 40% | 55% | 54% | 48% | 0.57 |
| Parental asthma | 37% | 32% | 28% | 26% | 41% | 0.82 |
| Parental education beyond high school | 52% | 45% | 51% | 67% | 45% | 0.79 |
| Household income ≥$15,000/year | 31% | 36% | 46% | 19% | 30% | 0.84 |
|  |  |  |  |  |  |  |
| FEV_1_ (liters)* | 2.06 (0.76) | 2.11 (0.80) | 2.24 (0.84) | 2.11 (0.76) | 1.86 (.47) | 0.54 |
| FEV_1_/FVC (%) | 85.5 (9.4) | 82.6 (10.0) | 85.7 (8.3) | 84.1 (6.2) | 81.5 (10.4) | 0.95 |
| Total IgE (IU/ml)^a^ | 163 (36-651) | 163 (51-622) | 114 (38-420) | 199 (46-712) | 190 (67-600) | 0.92 |
| STR(+) to: Mouse  Cockroach  Cat  Dust mite  Mold  STR to ≥1 allergen | 29%  29%  37%  43%  14%  81% | 18%  27%  30%  40%  14%  78% | 22%  30%  44%  37%  18%  67% | 21%  16%  32%  47%  11%  79% | 15%  30%  27%  48%  7%  71% | 0.17  0.77  0.58  0.64  0.48  0.18 |

Quintiles (and ranges) calculated using the combined cohort (San Juan and Hartford). Values shown are mean (SD) for continuous variables, except ^a^presented as median (IQR), analyzed as log10. STR=skin test reactivity. *FEV1 presented as absolute value due to lack of predicted values for Puerto Rican children.

**B) Hartford**

| **Quintile of mouse allergen level**  Range (ng/g)  N (%) | **Q1**  (.05-2.86)  8 (4.5) | **Q2**  (3.0-10.0)  15 (8.3) | **Q3**  (10.2-42.0)  38 (21.1) | **Q4**  (42.02-183.6)  59 (32.8) | **Q5**  (184-31,227)  60 (33.3) | **P for trend** |
| --- | --- | --- | --- | --- | --- | --- |
| Mean age (yrs) | 11.4 (2.7) | 9.4 (2.3) | 9.8 (3.0) | 9.3 (2.6) | 10.0 (3.1) | 0.63 |
| Male gender | 50% | 40% | 50% | 56% | 38% | 0.51 |
| Parental asthma | 50% | 43% | 41% | 44% | 47% | 0.77 |
| Parental education beyond high school | 25% | 20% | 26% | 31% | 25% | 0.78 |
| Household income ≥$15,000/year | 25% | 73% | 45% | 29% | 13% | **0.0001** |
|  |  |  |  |  |  |  |
| FEV_1_ (liters)* | 2.35 (0.83) | 1.91 (0.54) | 1.96 (0.69) | 1.88 (0.69) | 2.13 (0.76) | 0.11 |
| FEV_1_/FVC (%) | 84.9 (7.9) | 86.3 (4.6) | 86.6 (10.0) | 83.2 (9.6) | 85.0 (8.7) | 0.99 |
| Total IgE (IU/ml)^a^ | 78 (51-183) | 71 (31-159) | 125 (33-306) | 67 (28-251) | 43 (16-115) | **0.02** |
| STR(+) to: Mouse  Cockroach  Cat  Dust mite  Mold  STR to ≥1 allergen | 0%  0%  0%  67%  0%  50% | 0%  8%  8%  46%  0%  47% | 3%  15%  33%  36%  3%  58% | 2%  14%  7%  20%  0%  24% | 0%  18%  5%  18%  2%  28% | 0.74  0.22  0.11  **0.002**  0.76  **0.006** |

Quintiles (and ranges) calculated using the combined cohort (San Juan and Hartford). Values shown are mean (SD) for continuous variables, except ^a^presented as median (IQR), analyzed as log10. STR=skin test reactivity. *FEV1 presented as absolute value due to lack of predicted values for Puerto Rican children.
