# Supplementary material for: Mouse Allergen, Lung Function, and Atopy in Puerto Rican Children
Source: PLoS One. 2012 Jul 16;7(7):e40383. doi: 10.1371/journal.pone.0040383 (PMC3398035; doi:10.1371/journal.pone.0040383)
Supplement: Table S3 — Values shown are 1means or 2odds ratios and 95% confidence intervals, with P-values in parentheses. All allergens analyzed as log10. IgE analyzed as log10 and presented as percent increase/decrease. All models adjusted for age, sex, household income, and dust house levels of allergens. FEV1 adjusted additionally for height and height squared. (DOC) [file pone.0040383.s004.doc]

***Table S3. Multivariate analysis of mouse allergen level and selected outcomes in controls***

| **Predictors** | Pre-bronchodilator  FEV1 (mL)^1^ | Total  serum IgE^1^ | STR to at least  one allergen^2^ |
| --- | --- | --- | --- |
| **SAN JUAN** |  |  |  |
| Unadjusted (N) | 258 | 269 | 244 |
| Mus m 1 (ng/g) | -37.6 [-136.6;61.3] (0.46) | -0.6% [-18.7;21.5] (0.95) | 0.80 [0.59;1.09] (0.15) |
| Multivariate model (N) | 254 | 265 | 241 |
| Mus m 1 (ng/g) | **+40.8 [-6.6;88.1] (0.09)** | -2.9% [-20.6;18.8] (0.77) | 0.79 [0.57;1.09] (0.14) |
| Fel d 1 (μg/g) | -23.0 [-65;19] (0.28) | +16.5% [-2;39] (0.09) | 1.1 [0.8;1.4] (0.71) |
| Bla g (U/g) | -9.0 [-80;62] (0.80) | -15.6% [-37;14] (0.26) | 1.3 [0.8;2.1] (0.32) |
| Der p (μg/g) | -13.6 [-103;75] (0.76) | +6.5% [-27;55] (0.74) | 0.7 [0.4;1.2] (0.20) |
|  |  |  |  |
| **HARTFORD** |  |  |  |
| Unadjusted (N) | 179 | 168 | 180 |
| Mus m 1 (ng/g) | +58.9 [-67.2;185.0] (0.36) | **-26.1% [-42.8;-4.5] (0.02)** | **0.59 [0.40;0.88] (0.009)** |
| Multivariate model (N) | 148 | 138 | 149 |
| Mus m 1 (ng/g) | +0.8 [-56.6;58.2] (0.98) | **-25.4% [-44.9;0.5] (0.056)** | 0.69 [0.42;1.14] (0.15) |
| Fel d 1 (μg/g) | +109.3 [51;168] (0.0003) | -15.1% [-37;15] (0.29) | 0.9 [0.6;1.5] (0.76) |
| Bla g (U/g) | +6.3 [-58;70] (0.85) | +3.9% [-27;47] (0.83) | 0.9 (0.5;1.6] (0.74) |
| Der p (μg/g) | -73.1 [-155;9] (0.08) | -15.6% [-45;30] (0.44) | 0.9 [0.5;1.8] (0.82) |

Values shown are ^1^means or ^2^odds ratios and 95% confidence intervals, with P-values in parentheses. All allergens analyzed as log10. IgE analyzed as log10 and presented as percent increase/decrease. All models adjusted for age, sex, household income, and dust house levels of allergens. FEV1 adjusted additionally for height and height squared.
