# Supplementary material for: Mouse Allergen, Lung Function, and Atopy in Puerto Rican Children
Source: PLoS One. 2012 Jul 16;7(7):e40383. doi: 10.1371/journal.pone.0040383 (PMC3398035; doi:10.1371/journal.pone.0040383)
Supplement: Table S4 — a: Values shown are 1means or 2odds ratios and 95% confidence intervals, with P-values in parentheses. 3All children with STR to mouse also had STR to ≥ additional allergen. All allergens analyzed as log10. IgE analyzed as log10 and presented as percent increase/decrease. All models adjusted for age, sex, household income, dust house levels of allergens, and study site. FEV1 adjusted additionally for height and height squared. Table S4b, part A: Values shown are 1means or 2odds ratios and 95% confidence intervals, with P-values in parentheses. 3All children with STR to mouse also had STR to ≥1 additional allergen. All allergens analyzed as log10. IgE analyzed as log10 and presented as percent increase/decrease. All models adjusted for age, sex, household income, dust house levels of allergens, and disease status (case or control). FEV1 adjusted additionally for height and height squared. Unable to conduct multivariate analyses in Hartford. Table S4b, part B: Values shown are 1means or 2odds ratios and 95% confidence intervals, with P-values in parentheses. All allergens analyzed as log10. IgE analyzed as log10 and presented as percent increase/decrease. All models adjusted for age, sex, household income, dust house levels of allergens, and disease status (case or control). FEV1 adjusted additionally for height and height squared. Table S4b, part C: Values shown are 1means or 2odds ratios and 95% confidence intervals, with P-values in parentheses. 3All children with STR to mouse also had STR to ≥1 additional allergen. All allergens analyzed as log10. IgE analyzed as log10 and presented as percent increase/decrease. All models adjusted for age, sex, household income, dust house levels of allergens, study site, and disease status (case or control). FEV1 adjusted additionally for height and height squared. (DOC) [file pone.0040383.s005.doc]

***Table S4a. Multivariate analysis of mouse allergen level and selected outcomes in controls, by skin test reactivity to mouse***

| **Predictors** | Pre-bronchodilator FEV1 (mL)^1^ | Total serum IgE^1^ | STR to at least one allergen^2,3^ |
| --- | --- | --- | --- |
|  | **STR(+) to mouse** | | |
| Unadjusted (N) | 51 | 49 | n/a |
| Mus m 1 (ng/g) | +10.2 [-198.6;218.9] (0.92) | -7.6% [-42.7;49.0] (0.75) |  |
| Multivariate model (N) | 51 | 49 | n/a |
| Mus m 1 (ng/g) | -2.1 [-130.0;87.2] (0.70) | -11.0% [-44.9;43.9] (0.64) |  |
| Fel d 1 (μg/g) | -2.4 [-113;65] (0.60) | +48.8% [0.4;121] (0.048) |  |
| Bla g (U/g) | -51.6 [-209;106] (0.52) | -37.8% [-70;28] (0.20) |  |
| Der p (μg/g) | +96.8 [-89;283] (0.31) | +48.1% [-41;300] (0.40) |  |
|  | **STR(–) to mouse** | | |
| Unadjusted (N) | 300 | 305 | 313 |
| Mus m 1 (ng/g) | -21.1 [-105.0;62.7] (0.62) | **-29.2% [-40.7;-15.5] (0.0001)** | **0.59 (0.46;0.76] (0.0001)** |
| Multivariate model (N) | 271 | 276 | 283 |
| Mus m 1 (ng/g) | +23.2 [-17.3;63.7] (0.26) | -15.4% [-30.9;3.5] (0.10) | **0.67 [0.50;0.90] (0.009)** |
| Fel d 1 (μg/g) | +27.9 [-8;64] (0.13) | +0.9% [-16;21] (0.92) | 1.0 [0.8;1.3] (0.96) |
| Bla g (U/g) | +3.7 [-49;56] (0.89) | -11.6% [-32;15] (0.36) | 1.1 [0.7;1.6] (0.77) |
| Der p (μg/g) | -42.4 [-109;25] (0.21) | -16.7% [-41;17] (0.29) | 0.8 [0.5;1.3] (0.30) |

Values shown are ^1^means or ^2^odds ratios and 95% confidence intervals, with P-values in parentheses. ^3^All children with STR to mouse also had STR to ≥ additional allergen. All allergens analyzed as log10. IgE analyzed as log10 and presented as percent increase/decrease. All models adjusted for age, sex, household income, dust house levels of allergens, and study site. FEV1 adjusted additionally for height and height squared.

***Table S4b. Multivariate analysis of mouse allergen level and selected outcomes in all children, by skin test reactivity to mouse***

A) STR(+) to mouse, by study site

| **Predictors** | Pre-bronchodilator FEV1 (mL)^1^ | Total serum IgE^1^ | STR to at least one allergen^2,3^ |
| --- | --- | --- | --- |
|  | **SAN JUAN** | | |
| Unadjusted (N) | 108 | 107 | n/a |
| Mus m 1 (ng/g) | -3.7 [-138.3;130.9 ] (0.96) | -4.6% [-30.4;30.8] (0.77) |  |
| Multivariate model (N) | 108 | 107 | n/a |
| Mus m 1 (ng/g) | -0.4 [-88.0;87.3] (0.99) | -6.3% [-31.4;28.0] (0.68) |  |
| Fel d 1 (μg/g) | +14.9 [-65;94] (0.71) | +15.5% [-12;52] (0.31) |  |
| Bla g (U/g) | -41.6 [-168;85] (0.52) | -34.1% [-58;4] (0.07) |  |
| Der p (μg/g) | +74.3 [-92;241] (0.38) | +14.5% [-38;110] (0.66) |  |
|  | **HARTFORD** | | |
| Unadjusted (N) | 12 | 12 | n/a |
| Mus m 1 (ng/g) | +359 [-374;1093] (0.34) | -15.2% [-66.7;116.1] (0.73) |  |
|  |  |  |  |

Values shown are ^1^means or ^2^odds ratios and 95% confidence intervals, with P-values in parentheses. ^3^All children with STR to mouse also had STR to ≥1 additional allergen. All allergens analyzed as log10. IgE analyzed as log10 and presented as percent increase/decrease. All models adjusted for age, sex, household income, dust house levels of allergens, and disease status (case or control). FEV1 adjusted additionally for height and height squared. Unable to conduct multivariate analyses in Hartford.

B) STR(–) to mouse, by study site

| **Predictors** | Pre-bronchodilator FEV1 (mL)^1^ | Total serum IgE^1^ | STR to at least one allergen^2^ |
| --- | --- | --- | --- |
|  | **SAN JUAN** | | |
| Unadjusted (N) | 323 | 345 | 348 |
| Mus m 1 (ng/g) | +38.3 [-40.7;117.3] (0.34) | -8.7% [-22.9;8.2] (0.30) | 0.83 [0.65;1.05] (0.11) |
| Multivariate model (N) | 321 | 342 | 345 |
| Mus m 1 (ng/g) | **+47.1 [8.7;85.5] (0.016)** | -9.2% [-23.1;7.3] (0.26) | **0.79 [0.63;1.01] (0.07)** |
| Fel d 1 (μg/g) | -22.3 [-62;17] (0.27) | 0% [-16;18] (0.99) | 0.9 [0.7;1.2] (0.67) |
| Bla g (U/g) | -0.6 [-59;58] (0.98) | -20.3% [-38;2] (0.08) | 0.9 [0.6;1.3] (0.65) |
| Der p (μg/g) | -23.2 [-98;51] (0.54) | +5.7% [-24;46] (0.74) | 0.8 [0.5;1.3] (0.37) |
|  | **HARTFORD** | | |
| Unadjusted (N) | 335 | 329 | 337 |
| Mus m 1 (ng/g) | **+75.6 [-14.2;164.9] (0.09)** | -4.1% [-22.0;17.7] (0.69) | **0.80 [0.62;1.03] (0.09)** |
| Multivariate model (N) | 267 | 262 | 269 |
| Mus m 1 (ng/g) | **+42.6 (-5.9;91.1] (0.085)** | -5.7% [-27.0;21.9] (0.65) | 0.76 [0.53;1.08] (0.12) |
| Fel d 1 (μg/g) | +80.5 [34;127] (0.001) | -16.5% [-35;7] (0.15) | 0.8 [0.6;1.1] (0.22) |
| Bla g (U/g) | -7.1 [-58;44] (0.79) | -6.3% [-29;23] (0.64) | 0.9 [0.6;1.3] (0.58) |
| Der p (μg/g) | -100.0 [-164;-36] (0.002) | +23.6% [-12;73] (0.22) | 1.4 [0.9;2.3] (0.16) |

Values shown are ^1^means or ^2^odds ratios and 95% confidence intervals, with P-values in parentheses. All allergens analyzed as log10. IgE analyzed as log10 and presented as percent increase/decrease. All models adjusted for age, sex, household income, dust house levels of allergens, and disease status (case or control). FEV1 adjusted additionally for height and height squared.

**C) All children, by STR to mouse**

| **Predictors** | Pre-bronchodilator  FEV1 (mL)^1^ | Total  serum IgE^1^ | STR to at least  one allergen^2,3^ |
| --- | --- | --- | --- |
|  | **STR(+) to mouse** | | |
| Unadjusted (N) | 120 | 119 | n/a |
| Mus m 1 (ng/g) | -2.9 [-154.1;96.4] (0.65) | -3.0% [-22.8;37.3] (0.84) |  |
| Multivariate model (N) | 117 | 116 | n/a |
| Mus m 1 (ng/g) | -9.4 [-79.0;60.3] (0.79) | -9.1% [-32.6;22.6] (0.53) |  |
| Fel d 1 (μg/g) | -8.6 [-72;55] (0.79) | +15.5% [-12;51] (0.29) |  |
| Bla g (U/g) | -2.5 [-103;98] (0.96) | -33.5% [-57;3] (0.07) |  |
| Der p (μg/g) | +22.5 [-102;147] (0.72) | +12.6% [-35;94] (0.67) |  |
|  | **STR(–) to mouse** | | |
| Unadjusted (N) | 658 | 674 | 685 |
| Mus m 1 (ng/g) | +37.4 [-16.0;90.7] (0.17) | **-20.2% [-29.4;-9.8] (0.0003)** | **0.69 [0.59;0.81] (0.0001)** |
| Multivariate model (N) | 588 | 604 | 614 |
| Mus m 1 (ng/g) | **+28.3 [1.4;55.2] (0.039)** | -9.4% [-21.1;3.9] (0.16) | **0.77 [0.64;0.94] (0.009)** |
| Fel d 1 (μg/g) | +26.7 [-1;54] (0.06) | -6.5% [-19;7] (0.34) | 0.9 [0.7;1.1] (0.29) |
| Bla g (U/g) | +7.8 [-28;43] (0.67) | -12.5% [-27;5] (0.15) | 0.9 [0.7;1.2] (0.52) |
| Der p (μg/g) | -53.4 [-99;-8] (0.02) | +13.7% [-10;44] (0.28) | 1.1 [0.8;1.5] (0.68) |

Values shown are ^1^means or ^2^odds ratios and 95% confidence intervals, with P-values in parentheses. ^3^All children with STR to mouse also had STR to ≥1 additional allergen. All allergens analyzed as log10. IgE analyzed as log10 and presented as percent increase/decrease. All models adjusted for age, sex, household income, dust house levels of allergens, study site, and disease status (case or control). FEV1 adjusted additionally for height and height squared.
